# Anti-tumor efficacy of theliatinib in esophageal cancer patient-derived xenografts models with epidermal growth factor receptor (EGFR) overexpression and gene amplification

**DOI:** 10.18632/oncotarget.17243

**Published:** 2017-04-19

**Authors:** Yongxin Ren, Jianming Zheng, Shiming Fan, Linfang Wang, Min Cheng, Dongxia Shi, Wei Zhang, Renxiang Tang, Ying Yu, Longxian Jiao, Jun Ni, Haibin Yang, Huaqing Cai, Fang Yin, Yunxin Chen, Feng Zhou, Weihan Zhang, Weiguo Qing, Weiguo Su

**Affiliations:** ^1^ Department of Oncology Research, Hutchison MediPharma Limited, Shanghai, China; ^2^ Department of Pathology, Changhai Hospital, the Second Military Medical University, Shanghai, China; ^3^ Department of Chemistry, Hutchison MediPharma Limited, Shanghai, China

**Keywords:** esophageal cancer, patient derived xenograft models, EGFR targeted therapy, theliatinib

## Abstract

Targeted therapy is not yet approved for esophageal cancer (EC). In this study, we first evaluated EGFR gene and protein expression in 70 Chinese EC patient tumor samples collected during surgery. We then established 23 patient-derived EC xenograft (PDECX) models and assessed the efficacy of theliatinib, a potent and highly selective EGFR inhibitor currently in Phase I clinical study, in 9 PDECX models exhibiting various EGFR expression levels. Immunohistochemical analysis showed that 50 patient tumor samples (71.4%) had high EGFR expression. Quantitative PCR showed that eight tumors (11.6%) had *EGFR* gene copy number gain, and fluorescence *in situ* hybridization (FISH) revealed that four tumors had *EGFR* gene amplification. These results suggest that EGFR protein may be overexpressed in many EC tumors without gene amplification. Also detected were rare hot-spot mutations in *EGFR* and *PIK3CA*, whereas no mutations were found in *K-Ras* or *B-Raf*. Theliatinib exhibited strong antitumor activity in PDECX models with high *EGFR* expression, including remarkable tumor regression in two PDECX models with both *EGFR* gene amplification and protein overexpression. However, the efficacy of theliatinib was diminished in models with *PI3KCA* mutations or FGFR1 overexpression in addition to high EGFR expression. This study demonstrates that theliatinib could potentially benefit EC patients with high EGFR protein expression without mutations or aberrant activities of associated factors, such as *PI3KCA* or FGFR1.

## INTRODUCTION

Esophageal cancer (EC) is the eighth most common cancer worldwide and ranks in the top 5 in China in terms of patient cases and mortalities [1, 2]. The 2012 Chinese cancer registry annual report shows that EC accounts for nearly 10% of all cancer deaths. Large numbers of EC patients were generally diagnosed in an advanced stage and had poor prognosis. Patients with unresectable or metastatic EC are typically treated with chemotherapy using a combination of 5-fluorouracil (5-FU) and cisplatin and have a median survival of less than one year [3]. There are limited salvage options for patients with refractory EC [4] and targeted therapies are not yet available for EC. Therefore, there is great scope and need for novel targeted therapeutic options for EC.

Overexpression of EGFR is commonly found in EC and is associated with poor prognosis [5]. Unlike colon or lung cancers, *K-Ras* mutations are less frequent in EC [6]. This suggests that inhibition of EGFR pathway maybe therapeutically beneficial to EC patients with EGFR overexpression. However, clinical trials with anti-EGFR antibodies or first generation tyrosine kinase inhibitors (TKIs) in advanced EC patients have been disappointing [7, 8, 9], partly due to lack of prospective patient selection. For example, in the phase III COG trial conducted in mainly esophageal adenocarcinoma patients, the overall survival rates for gefitinib and placebo controls were comparable (3.73 months for gefitinib and 3.67 months for placebo), although there were slight improvements in progression free survival, disease control rate and quality of life [10]. Subsequently, a TRANS-COG trial that was prospectively as part of the COG trial demonstrated that gefitinib significantly enhanced overall survival in patients carrying *EGFR* gene amplification (HR = 0.19, *p* = 0.007) and EGFR gene copy number gain (HR = 0.53, *p* = 0.042) [11]. A recent pre-clinical study showed that esophageal tumor cell lines with high polysomy of *EGFR* were sensitive to gefitinib [12]. These findings suggested that EGFR directed therapy could be beneficial to esophageal cancer patients with high EGFR protein expression or gene copy number. Although the TRANS-COG trial investigated the relationship between *EGFR* gene expression and anti-tumor response of gefitinib, EGFR protein expression was not reported. Therefore the correlation between EGFR protein expression and response to EGFR-TKI is not yet clear.

Theliatinib is a novel EGFR tyrosine kinase inhibitor that is currently being evaluated in phase I clinical trial in China (NCT02601248). We conducted a pre-clinical study to evaluate the anti-tumor activity of theliatinib in a panel of patient derived esophageal cancer xenograft (PDECX) models to determine the association between anti-tumor activity of theliatinib and different levels of EGFR expression in EC tumors.

## RESULTS

### EGFR gene amplification and protein expression in tumor tissues from Chinese esophageal cancer patients

**Figure 1:**
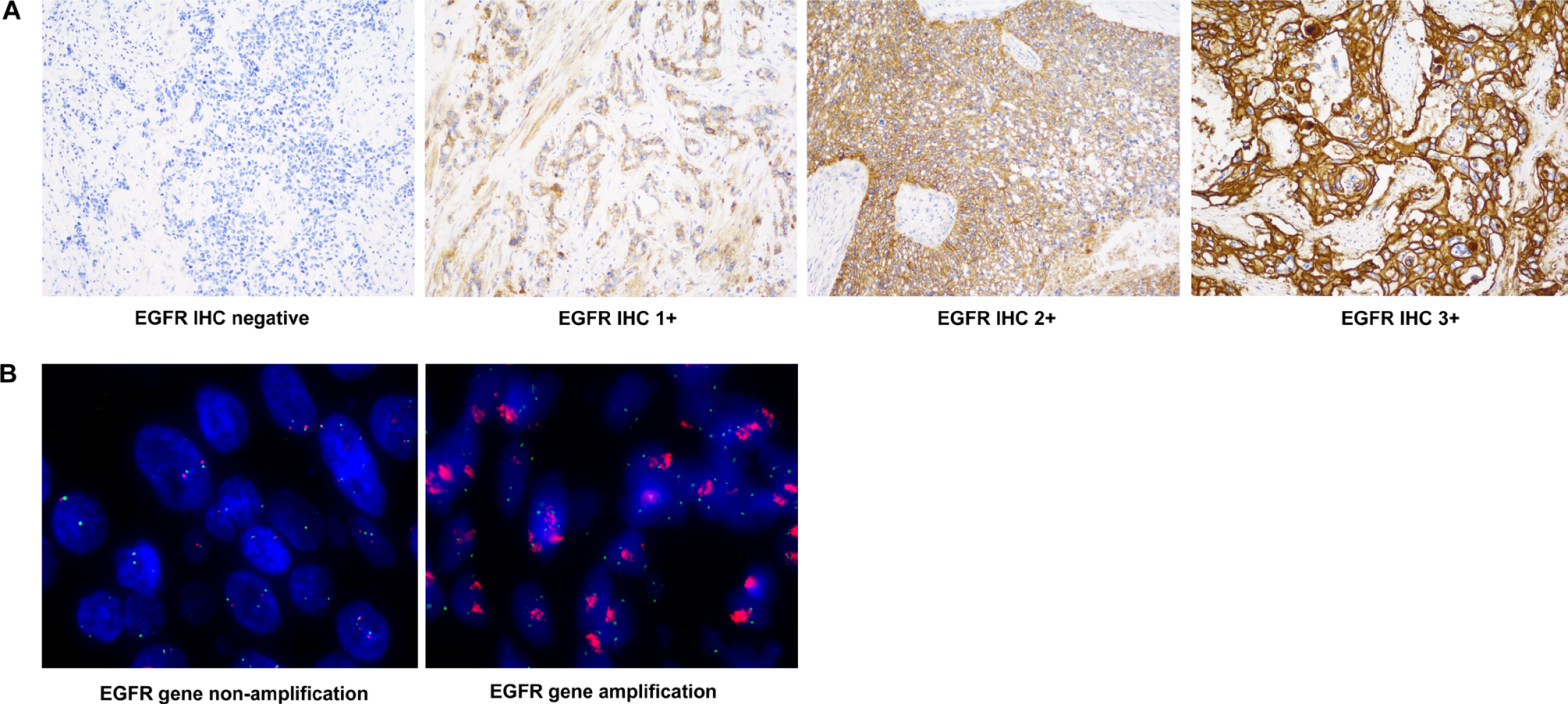
Representative images of EGFR IHC and FISH (**A**) EGFR IHC images (400×) for 0, 1+, 2+ and 3+ scores are shown. (**B**) EGFR FISH staining images (1000×) tumor samples with or without *EGFR* gene amplification are shown.

First, we evaluated EGFR expression status by immunohistochemistry (IHC) in 70 tumor specimens from Chinese EC patients, including 65 squamous-cell carcinoma, 2 adenocarcinoma, 2 small-cell carcinoma and 1 sarcoma (Supplementary Table 1). Sixty-four specimens showed positive EGFR expression with 50 demonstrating high EGFR expression (H score ≥ 200; Table 1). Further we conducted quantitative PCR analysis and found in 8 out of 69 specimens showing *EGFR* gene copy number gain (≥ 2.0; Table 1). Four of the eight specimens showed amplified *EGFR* gene (*EGFR* gene copy number/*CEP 7* ratio ≥ 2.0) by FISH analysis (Figure 1). Interestingly, all 8 specimens with *EGFR* gene copy number gain demonstrated an EGFR H score ≥ 290 (Supplementary Table 1). Also, 13 specimens that did not show *EGFR* copy number gain demonstrated EGFR H scores > 290 (Supplementary Table 1). This indicated that some tumors without *EGFR* gene copy number gain could have high EGFR protein expression.

**Table 1:** EGFR expression in treatment-naive Chinese esophageal cancer patients

Histopathology (*n* = 70)		EGFR IHC H score (*n* = 70)				EGFR copy number gain (*n* = 69)	EGFR hot spot mutation (*n* = 66)
squamous	others	0	10~90	100~190	200~300	by qPCR	by Sanger sequencing
65 (92.9%)	5 (7.1)	6 (8.6%)	2 (2.9%)	12 (17.1%)	50 (71.4%)	8 (11.6%)	1 (1.5%)

### Gene mutations of *EGFR* and downstream targets

Next, we successfully performed DNA extraction and Sanger sequencing in 66 out of 70 samples to determine hot spot mutations in *EGFR*, *PIK3CA*, *K-Ras* and *B-Raf* genes. The profiling data for the 70 tumor specimens are provided in Supplementary Table 1. A substitution was identified in the kinase domain (exon 21; c.2549 A>T, H850L) of the *EGFR* (Table 1). However, the L858 mutation or frame shift mutations were not detected in the patient tumor samples (Table 1). Further, as shown in Supplementary Table 1, synonymous single nucleotide polymorphisms (SNPs) were detected in 18 patient samples in exon 20 (c.2361G>A, p.Q787Q) and 1 patient in the exon 19 (c.2235 G>A, p.K745K) of the EGFR. A *PIK3CA* hot-spot mutation (E542K) was detected in exon 9 in 1 patient sample (Table 1). No *B-Raf* or *K-Ras* hot-spot mutation was detected in this study (Table 1).

### Establishment of PDECX models

**Figure 2:**
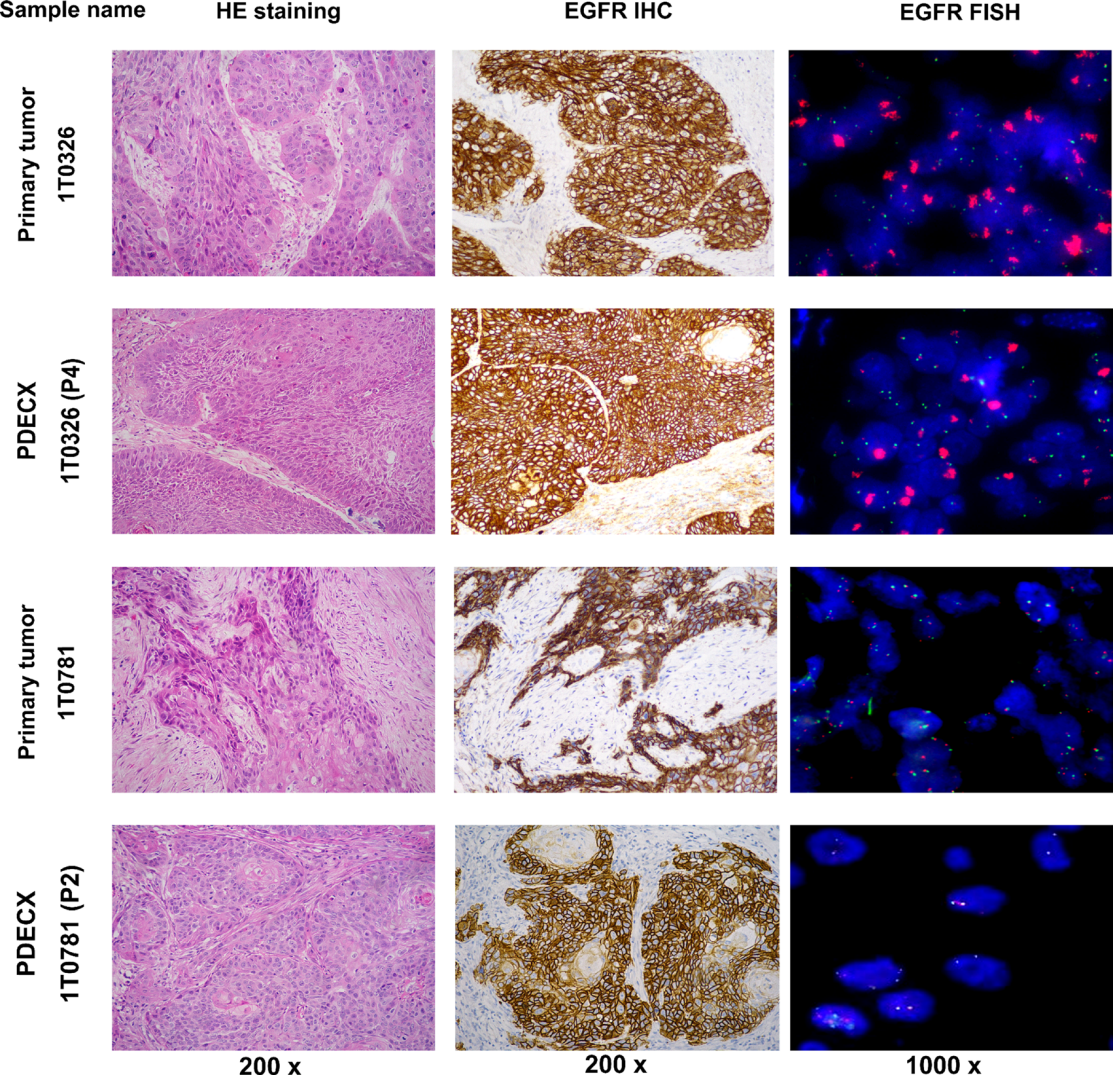
H&E, EGFR IHC and FISH staining of PDECX models and corresponding primary patient tumor specimens Images of H&E (200×), EGFR IHC (200×) and FISH (1000×) for PDECX 1T0326 (P4) and PDECX 1T0781 (P2) models are shown with the corresponding human primary tumor tissue.

As of July 2014, 54 fresh EC samples were implanted subcutaneously into NOD-SCID mice, of which 23 (42.6%) grew for 3 consecutive passages (P3). Fourteen of the 23 well-growing PDECX models, including 11 with high EGFR expression (H score ≥ 200) were successfully profiled. Finally, top 9 of the 14 PDECX models (Table 2) with a broad range of EGFR expression levels (H score = 15~300) were selected to assess the correlation between EGFR expression levels and theliatinib sensitivity. Then, we compared the histological and molecular characteristics between PDECXs and the corresponding patient tumor samples and found no significant changes during the serial tumor tissue passages, indicating that the PDECX models closely represented the human tumors. Analyses of two representative models, PDECX1T0326 and PDECX1T0781 with their corresponding primary tumors are shown in Figure 2.

**Table 2:** Profiling of 14 PDECX models established in this study

Model ID	Histopathology	EGFR IHC H score	*EGFR* GCN by qPCR	*EGFR* mutation	*PIK3CA, K-Ras* and *B-Raf* mutation
1T0326	ESCC	290	11.7	No	No
1T0950	ESCC	280	27.4	No	No
1T0472	ESCC	300	1.1	No	PIK3CA^E542K^
1T1315	ESCC	295	2.3	No	No
1T0327	ESCC	285	1.1	No	No
1T0781	ESCC	270	1.6	No	No
1T0994	ESCC	230	1.8	Q787Q	No
1T0474	ESCC	180	1.4	No	No
1T0773	ESCC	15	0.8	No	No
1T1035	ESCC	290	2.3	No	No
1T1058	ESCC	295	2.0	Q787Q	No
1T1230	ESCC	280	2.1	No	No
1T1061	ESCC	280	2.1	No	No
1T0857	ESCC	190	2.0	No	No

The top 9 models were used to determine anti-tumor activity of theliatinib.

### Theliatinib is a highly selective and potent ATP-competitive inhibitor of EGFR

**Figure 3:**
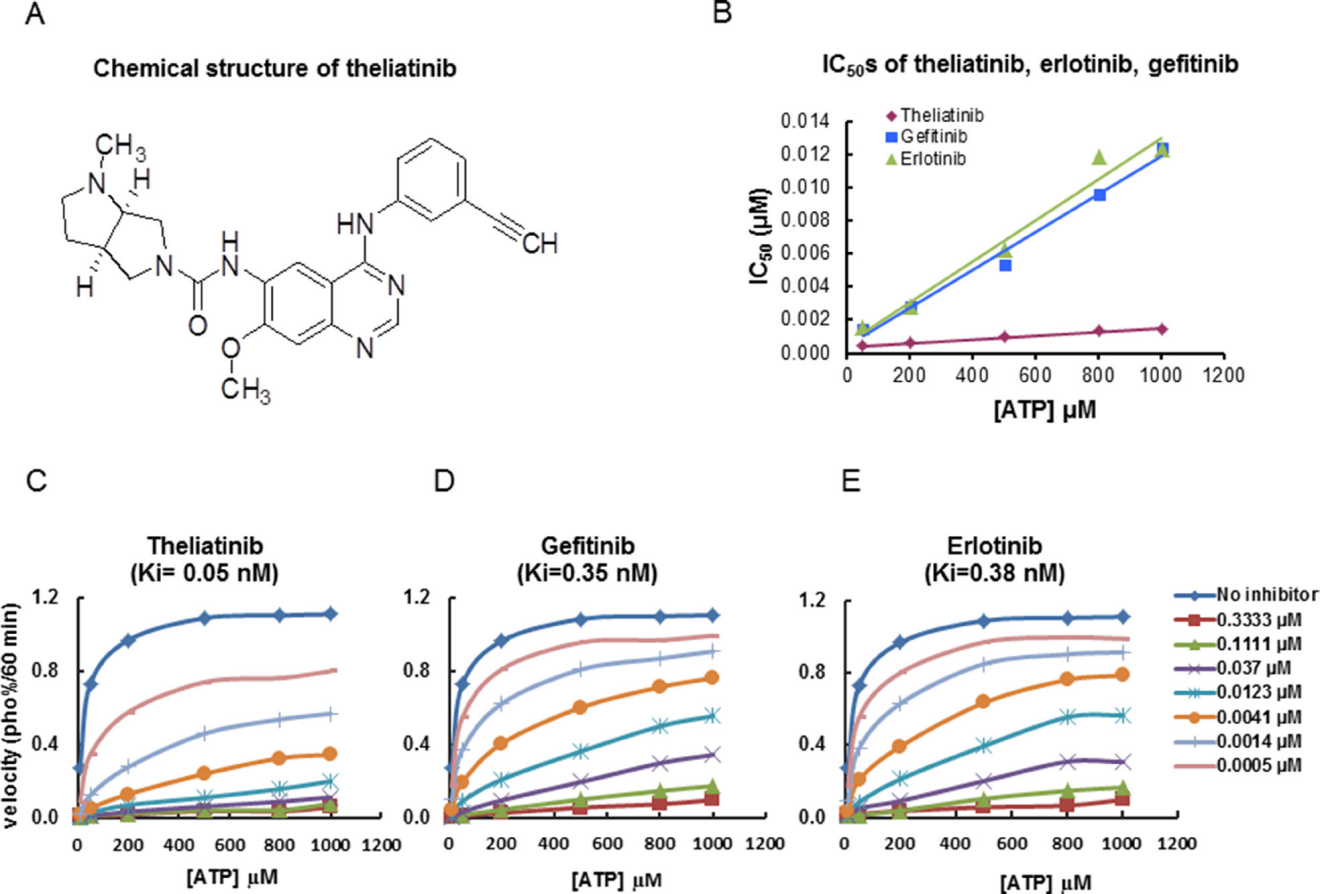
Chemical structure of theliatinib and its enzyme kinetics on EGFR inhibition (**A**) Chemical structure of theliatinib. (**B**) IC_50_ values of theliatinib, gefitinib and erlotinib were calculated by determining inhibition of wild type EGFR kinase in presence of different ATP concentrations (10~1000 μM) as shown. At all tested ATP concentrations, theliatinib showed lower IC_50_ compared with gefitinib or erlotinib. (**C**–**E**) The effects of different concentrations of theliatinib, gefitinib and erlotinib (0~0.3333 μM) on the reaction velocity of EGFR kinase at different ATP concentrations (10~1000 μM) are shown. The Ki values of theliatinib, erlotinib and gefitinib were calculated using Michaelis-Menten equation in Graphpad Prism software.

The chemical structure of theliatinib is shown in Figure 3A [13]. Theliatinib is a highly potent EGFR inhibitor with 3~7 fold greater potency than erlotinib or gefitinib at both the enzyme and the cell level (Figure 3B and Table 3). The enzyme kinetics studies demonstrated that theliatinib, gefitinib or erlotinib were all ATP-competitive inhibitors with Ki values 0.05, 0.35 and 0.38 nM, respectively against the wild type EGFR (Figure 3C–3E). The IC_50_ of theliatinib against EGFR and EGFR T790M/L858R mutant was 3 and 22 nM, respectively (Supplementary Table 2). Also, theliatinib demonstrated 50 fold greater selectivity for EGFR compared to 72 other kinases (Supplementary Table 2) suggesting that it was a highly selective EGFR inhibitor.

**Table 3:** Theliatinib inhibits EGFR phosphorylation and cell survival in tumor cells with wild-type EGFR

	Theliatinib (IC_50_, μM)	Erlotinib (IC_50_, μM)
**EGF stimulated EGFR phosphorylation**		
A431	0.007 ± 0.002, *n* = 3	0.026 ± 0.005, *n* = 3
**Tumor cell survival**		
A431(epidermoid)	0.8	2.4
H292 (lung)	0.058	0.341
FaDu (pharynx)	0.354	1.2

### *In vivo* anti-tumor activity of theliatinib in multiple PDECX models

Nine PDECX models were selected to evaluate *in vivo* anti-tumor activity of theliatinib, including 7 with high EGFR expression (H score > 200), 2 (PDECX 1T0326 and PDECX 1T0950) with simultaneous *EGFR* gene amplification and high EGFR protein expression, 1 with medium EGFR expression (PDECX 1T0474, H score = 180) and 1 with low EGFR expression (PDECX 1T0773, H score = 15). We selected 15 mg/kg/day theliatinib and 20 mg/kg/day gefitinib doses for mice that were similar to those achieved in the clinics.

**Figure 4:**
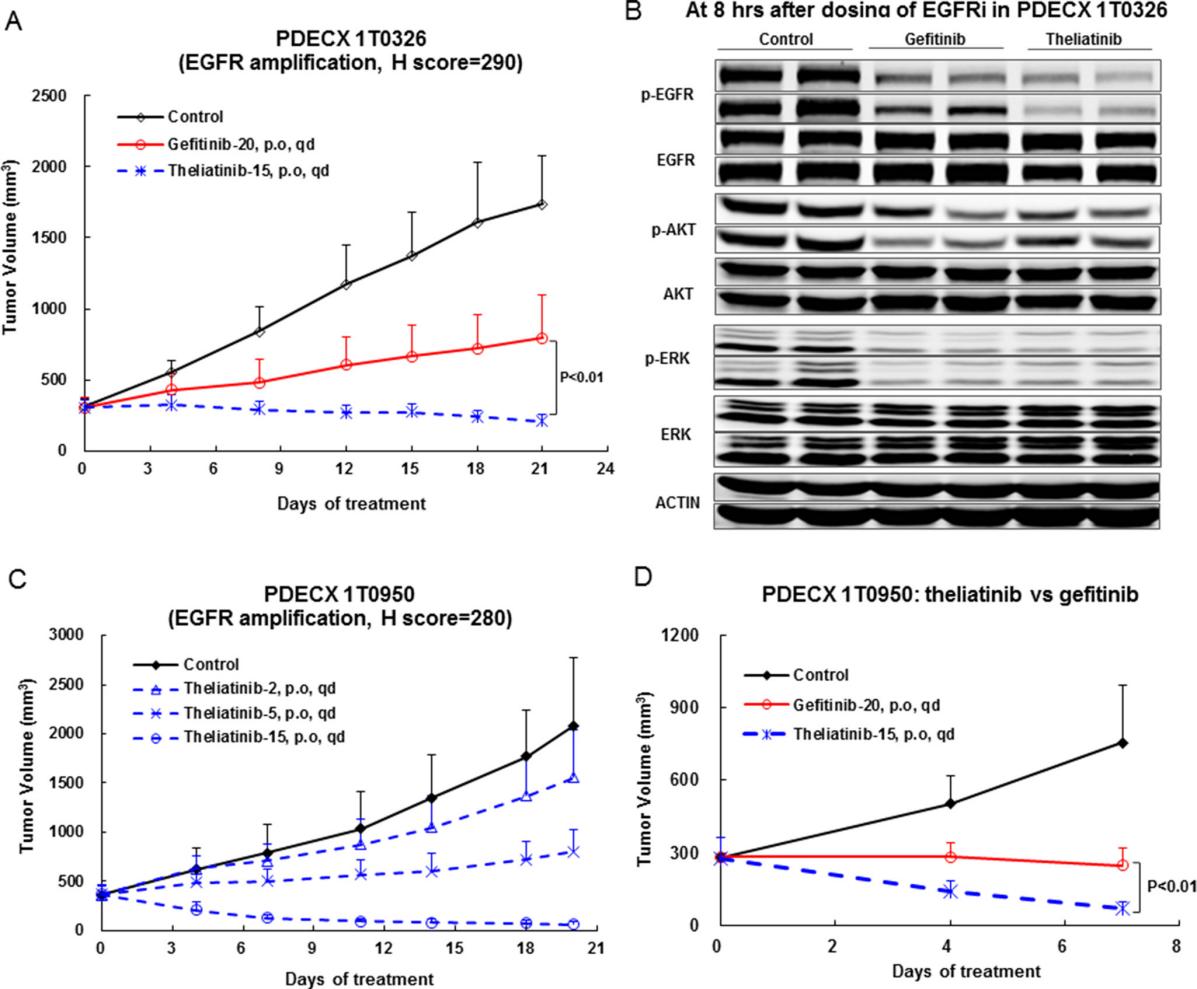
Anti-tumor efficacy of theliatinib in PDECX models with *EGFR* gene amplification and overexpression (**A**) Comparison of anti-tumor effects of theliatinib and gefitinib in PDECX 1T0326 model is shown. (**B**) Western blot analysis demonstrating the phosphorylation status of EGFR, AKT and ERK in patient tumor xenografts 8 h after oral administration of theliatinib or gefitinib to nude mice is shown. Western blot analysis was performed on subcutaneous tumors (4 mice/group). (**C**) Theliatinib attenuates tumor growth in PDECX 1T0950 model in a dose-dependent manner (2, 5 and 15 mg/kg/day). (**D**) Theliatinib demonstrates stronger anti-tumor activity than gefitinib in the PDECX 1T0950 model. The subcutaneous tumor volume was measured and calculated. Y-axis represented the volume of the tumor (Mean ± SD), and X-axis represents days after first dose being administered.

**Figure 5:**
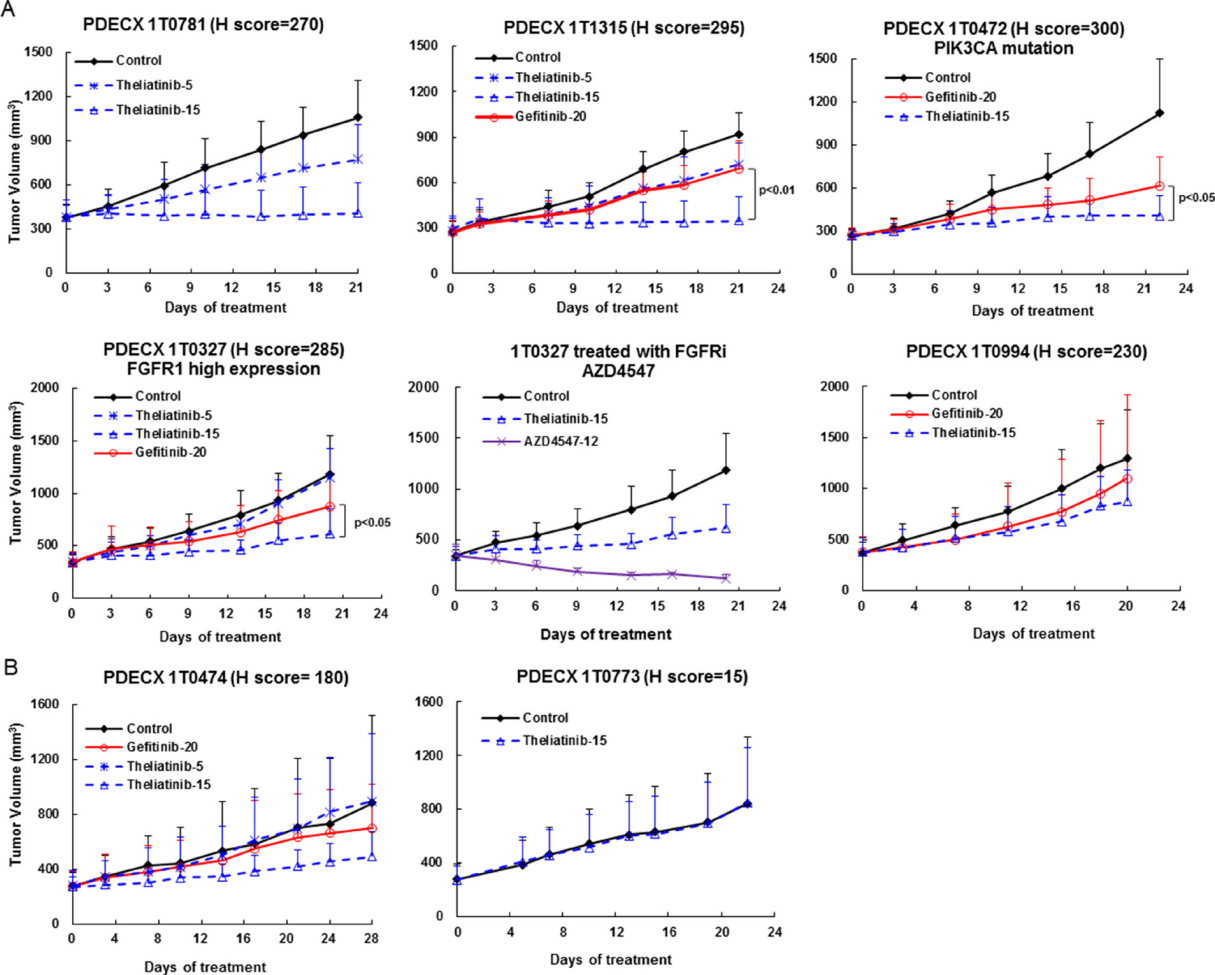
Anti-tumor efficacy of theliatinib in PDECX models without *EGFR* gene amplification (**A**) Anti-tumor effects of theliatinib (5 or 15 mg/kg/day), gefitinib (20 mg/kg/day) are compared against negative control in PDECX models 1T0781, 1T1315, 1T0472, 1T0327 and 1T0994 with EGFR H score > 200. Also shown is 1T0327 with AZD4547 (FGFR inhibitor) compared to theliatinib treatment. (**B**) Theliatinib shows low or moderate anti-tumor activity in PDECX models 1T0474 and 1T0773 with EGFR H score < 200.

We observed that the two PDECX models with *EGFR* gene amplification (PDECX1T0326 and PDECX1T0950) were most sensitive to theliatinib treatment demonstrating tumor regression of 32% and 75%, respectively, at the end of study (Figure 4 and Table 4). The PDECX1T0326 model demonstrated significant inhibition of phosphorylation of EGFR (p-EGFR) and its downstream targets, AKT and ERK (p-AKT and p-ERK) as shown in Figure 4B. In the PDECX1T0950 model, a dose response (2, 5 and 15 mg/kg) was observed (Figure 4C), suggesting that anti-tumor activity was dependent on the level of inhibition of EGFR pathway activation. Gefitinib also displayed anti-tumor activity in these two models with *EGFR* gene amplification (Figure 4A and Figure 4D). The 20 mg/kg/day dose of gefitinib administered to nude mice was similar to the 500 mg/day dose used in the phase III COG trial. In most PDECX models, the anti-tumor effect of 15 mg/kg theliatinib was significantly better than 20 mg/kg gefitinib (Figure 4A, 4D and Figure 5).

**Table 4:** Anti-tumor activity of theliatinib in PDECX models

PDECXModel ID	EGFR H score	EGFR gene amplification	Others	% TGI (% regression)		
				Theliatinib 15 mg/kg	Gefitinib 20 mg/kg	*p* value (theliatinib vs gefitinib)
1T0326	290	Yes	-	106.8** (31.6)	65.6**	< 0 .01
1T0950	280	Yes	-	144.4** (75.3)	107.3** (12.1)	< 0.01
1T0781	270	No	-	95.9**	ND	ND
1T1315	295	No	-	91.8**	34.5*	< 0.01
1T0472	300	No	PIK3CA^E542K^	83.9**	60.3**	< 0.05
1T0327	285	No	FGFR1 OE	67.4**	36.6	< 0.05
1T0994	230	No	EGFR Q787Q	46.3*	21.8	> 0.05
1T0474	180	No	-	63.6	29.5	> 0.05
1T0773	15	No	-	−2.7	ND	ND

TGI: tumor growth inhibition. OE: over-expression. ND: not determined. **p < 0.01; *p < 0.05 versus control group.

Generally, the anti-tumor efficacy of theliatinib correlated with the level of EGFR expression in PDECX models (Figure 5 and Table 4). Robust efficacy was observed in PDECX1T0781 and PDECX1T1315 that demonstrated high EGFR expression (Figure 5A and Table 4). However, the efficacy of theliatinib was attenuated in PDECX1T0472 and PDECX1T0327 that also had high EGFR expression, probably due to other gene alterations, such as *PIK3CA* mutation in PDECX1T0472 and FGFR1 overexpression in PDECX1T0327 (Figure 5A and Table 4). In fact, the PDECX1T0327 model demonstrated rapid tumor regression upon treatment with AZD4547 (12 mg/kg), a selective FGFR inhibitor, suggesting that FGFR may be a stronger driver in this tumor than EGFR. These results suggested that when multiple pathways are aberrantly activated in tumors, combination therapy would be necessary. In models with medium or low EGFR expression (PDECX1T0474 and PDECX1T0773), theliatinib demonstrated low to moderate efficacy (Figure 5B and Table 4).

## DISCUSSION

In the present study, 93% (65/70) of Chinese esophageal tumor samples were squamous cell carcinoma (ESCC) in contrast to mostly adenocarcinoma in Caucasian population [14]. ESCC tumors are mostly situated in the upper two thirds of the esophagus and associated with smoking and alcohol, salty foods and nitrosamine in foods. In contrast, esophageal adenocarcinoma (EAC) tumors are located almost exclusively in the lower third of the esophagus and esophagogastric junction and are associated with Barrett's metaplasia and chronic gastro-esophageal reflux [14, 15]. Apart from the histological and epidemiological distinctions, ESCC and EAC differ in molecular features [16]. These differences suggest that the two cancer subtypes have distinct molecular pathogenesis and genetic alterations. However, the COG and TRANS-COG trials demonstrated that the tumor response to EGFR-TKI correlates with EGFR status rather than histological subtypes [10, 11]. The preclinical results presented in our study further demonstrate that tumors with high EGFR expression with or without *EGFR* gene copy number gains result in sensitivity to theliatinib treatment. Therefore, our data demonstrates that theliatinib would be clinically beneficial to esophageal cancer patients with high EGFR expression.

Since the anti-tumor effect of theliatinib correlated with EGFR expression levels in the PDECX models, patient selection is important for beneficial outcomes. The two PDECX models with *EGFR* gene amplification were most sensitive to theliatinib treatment with rapid and robust tumor regression. The PDECX models without *EGFR* amplification but with high EGFR expression (IHC H score = 270~300) also responded strongly to theliatinib with tumor growth inhibition of 83~96%. In contrast, the PDECX models with low EGFR expression (IHC H score < 200) had low to moderate effect upon theliatinib treatment. Therefore, our study indicates that tumors with *EGFR* gene amplification and high EGFR expression may demonstrate improved object response rate (ORR), whereas tumors with high EGFR expression without *EGFR* gene amplification may demonstrate improved progression free survival (PFS) with or without ORR. Based on the preclinical findings of theliatinib, we propose that esophageal cancer patients with high EGFR levels (IHC H score ≥ 270) should potentially benefit from theliatinib treatment.

The presence of EGFR activating mutations is an established predictive biomarker in non-small cell lung cancer (NSCLC) with in-frame deletions of exon 19 (E746_A750) and L858R substitution in exon 21 accounting for > 90% of all activating mutations [17]. However, these mutations are distinctly less common in esophageal cancer [18]. In the present study, exon 19 deletions and L858R mutation in exon 21 were not detected in any of the 70 patients. However, a novel EGFR H850L mutation in exon 21 was found in 1 patient and the biological significance of this mutation needs to be determined in future. Further, we found that in 27% (18/66) of cases, an EGFR SNP at codon 787 of exon 20 was present, similar to a previous finding in Japanese ESCC patients in which Q787Q SNP was reported to be associated with decreased overall survival in the patients that received chemoradiotherapy [19]. Interestingly, Q787Q SNP was also identified in head and neck cancer cell lines and patient samples and was associated with higher sensitivity to gefitinib [20]. In our anti-tumor studies, one PDECX model (1T0994, H score 230) that carried Q787Q SNP was insensitive to theliatinib. Therefore, the molecular details between Q787Q SNP and response to EGFR-TKIs need to be investigated further. Overall, EGFR hot-spot mutations were rare in our study.

It is also well recognized that lung cancer cells carrying wild-type EGFR are less sensitive to EGFR TKIs than lung cancer cells with EGFR activating mutations [21, 22]. This was responsible for failure of the first generation TKIs, gefitinib or erlotinib in lung cancer patients with wild type EGFR in earlier clinical trials. In comparison to erlotinib or gefitnib, theliatinib showed much stronger binding affinity to wild type EGFR and was more difficult to be replaced by ATP. This unique feature may result in better target engagement for theliatinib compared to erlotinib or gefitinib, leading to stronger anti-tumor activity in tumors with wild type EGFR activation due to gene amplification or protein overexpression.

Our study also demonstrates that aberrant function of other receptor tyrosine kinase receptors or somatic mutations in down-stream targets such as FGFR overexpression or *PIK3CA* mutation can influence drug sensitivity of EC to EGFR TKIs. The frequency of *PIK3CA* mutations in EC varies from 0% to 21% [23]. However, the predictive or prognostic role of the *PIK3CA* mutation in EC remains unclear. In our study, one EC sample with a *PIK3CA* E542K mutation on exon 9 and the corresponding PDECX1T0472 showed moderate sensitivity to theliatinib treatment with 84% TGI, which was lower than two other PDECX models with similar EGFR H scores, 1T0781 and 1T1315, without any *PIK3CA* mutation. This suggested that the *PIK3CA* hot-spot mutations may attenuate the sensitivity to EGFR inhibitors. Therefore, it would be interesting to investigate anti-tumor activity of theliatinib in combination with a PI3K inhibitor in the future. FGFR1 gene amplification was reported in ESCC with a frequency of 6% to 21% [24] and protein overexpression in 17% of ESCC [25]. However, the therapeutic potential of FGFR1 targeted therapy in ESCC has not been fully evaluated. The PDECX model 1T0327 with overexpression of both EGFR and FGFR1 protein showed only modest tumor growth inhibition to theliatinib treatment suggesting that FGFR1 overexpression could diminish theliatinib efficacy. Interestingly, treatment with a FGFR inhibitor AZD4547 induced rapid tumor regression, suggesting that FGFR1 overexpression was driving the tumor growth in the specific model. This is also the first report of anti-tumor activity of a FGFR TKI in a PDECX model with high FGFR1 expression. We propose to test the efficacy of FGFR TKI either alone or in combination with EGFR TKI to evaluate its therapeutic benefits in ESCC.

There are some limitations of our study. Firstly, the sample size was relatively small and may have inherent bias. Secondly, although Sanger sequencing is a cost-effective method to detect gene mutations, it has several drawbacks such as low sensitivity that may result in not detecting novel mutations. Thirdly, *in vivo* theliatinib target inhibition study was carried out at 8h after treatment with 15 mg/kg theliatinib (Figure 4B). We propose further investigating the target inhibition effect at 24 h to clearly establish the relationship between target inhibition and anti-tumor activity.

In summary, we observed high EGFR protein levels in majority of Chinese EC patient samples that we analyzed. Comparatively, *EGFR* gene amplification was less prevalent in these patients. Theliatinib, a novel EGFR TKI with strong affinity to wild type EGFR protein demonstrated dose-dependent anti-tumor activity in a panel of PDECX models with a generally good correlation between EGFR H score and tumor growth inhibition. Furthermore, aberrant activation or gene mutations of other targets such as PI3K and FGFR diminished the anti-tumor activity of the EGFR TKIs, especially, theliatinib. In conclusion, our data suggests that thelaitinib would be beneficial for patients that have high EGFR expression thereby proper patient selection strategy would result in enhanced drug efficacy. Therefore, in phase IB trials, it would be worthwhile to analyze the ORR, DCR and PFS parameters in EC patients with high EGFR protein expression (IHC H score ≥ 270, with or without *EGFR* gene amplification).

## MATERIALS AND METHODS

### Theliatinib preparation for *in vitro* and *in vivo* studies

Theliatinib (3a*R*,6a*R*)-*N*-(4-(3-ethynylphenyl-amino)-7-methoxyquinazolin-6-yl)-1-methyl-hexahydropyrrolo [3,4-*b*]pyrrole-5(1*H*)-carboxamide; molecular weight 442.21; Figure 3A) was synthesized by Hutchison MediPharma Limited (HMP). Theliatinib was prepared as a 10 mmol/L stock solution in DMSO and diluted in appropriate assay media for *in vitro* assays. Theliatinib was suspended in aqueous 0.5% Sodium Carboxymethyl Cellulose (CMC-Na) and stored at 4°C for *in vivo* studies. Gefitinib, Erlotinib and AZD4547 were provided by Department of Chemistry, HMP.

### Chinese esophageal cancer patients in this study

Seventy esophageal tumor specimens from newly diagnosed patients were collected during surgical resection from the Shanghai Biobank Network of Common Human Tumor Tissue at the Changhai hospital, Shanghai, China. Two additional surgical EC samples used for PDECX models establishment were provided by Renji Hospital, Shanghai. Prior written informed consent was obtained from all patients.

### PDECX models and anti-tumor efficacy studies

Seven- to nine week old NOD-SCID (NOD.CB17-Prkde<scid>/JSlac) immunodeficient or BALB/cASlac-nu/nu male or female mice were obtained from Shanghai SLAC Laboratory Animal Co. Ltd or Shanghai LingChang BioTech Co. Ltd. Fresh tumor specimens from newly diagnosed patients were collected during surgery and separated into three parts for the following: (1) To prepare formalin fixed paraffin embedded (FFPE) sections; (2) For snap freezing in liquid nitrogen for DNA extraction and sequencing and (3) For subcutaneous implantation into NOD-SCID mice (P0) and subsequent passages in additional NOD-SCID or nude mice once the tumor size reached 800~1500 mm^3^. After several consecutive *in vivo* passages, the PDECX models (P3~P7) were used to evaluate the anti-tumor efficacy of theliatinib or gefitinib or AZD4547. All experiments in animals were performed in accordance with protocols approved by the Hutchison MediPharma Limited Animal Care and Use Committee (HMPLACUC).

When the average tumor volume reached the 250~500 mm^3^, mice were randomly divided into different experimental groups. Tumor-bearing mice were daily administered oral doses of test compounds (Theliatinib in 0.5% CMC-Na; Gefitinib in 0.5% Tween-80 and AZD4547 in 1% Tween-80 (tested only in the PDECX 1T0327 model)) or vehicle control. Body weight and tumor size of all mice was measured twice or thrice a week. Tumor volumes (TV) were calculated by measuring two perpendicular diameters with calipers (formula: TV = (length × width^2^)/2). Tumor growth inhibition (TGI) was calculated using the formula TGI= [1-(V*t* -V*0*)*drug treated* / (V*t* -V*0*)*vehicle control*]×100%. Statistical significance was determined by Student's *t* test and *p* < 0.05 was considered statistically significant.

### Hematoxylin and Eosin (H&E) and EGFR- IHC staining and scoring

Tissues from EC patients were harvested and fixed in 10% buffered formalin for 24 h within 30 minutes after resection. Then, 4 μm tissue sections were cut and paraffinized before storage. H&E staining were performed following the routine procedures [26] and finally, diagnosed by pathologists at the Changhai hospital. IHC staining on patient tumor sections was performed with EGFR PharmDx (DAKO, K1494) on DAKO auto-stainer Link48 according to the manufacturer's instructions. Briefly, 4 μm tissue sections were prepared and dried for 1h at room temperature and then placed in a 56~60°C incubator for 1h. After deparaffinization and rehydration [26], sections were incubated with proteinase K solution for 5 minutes. Endogenous peroxidase activity was blocked with 3% hydrogen peroxide for 5 minutes and washed with the buffer followed by incubation with mouse monoclonal anti-human EGFR antibody (clone2–18C9c) for 30 minutes. Then, the sections were incubated with secondary goat anti-mouse antibody conjugated with horseradish peroxidase for 30 minutes followed by diaminobenzidine (DAB) substrate chromogen solution for 10 minutes. Monoclonal mouse IgG1 antibody was used as negative control. In each IHC staining run, a control slide provided by EGFR pharmDx^™^ kit with IHC 0 and 2+ was also included. The tumor sections from PDECX samples were manually treated with EGFR antibody (Cell Signaling Technology, Cat#4267) followed by biotinylated secondary antibody and the DAB chromogen.

The percentage of tumor cells with positive staining were reviewed and scored using a four-tier system on a scale of 0, 1+, 2+ or 3+, and H score was calculated as follows: H score=100 × [1 × (% of 1+ cells) + 2 × (% of 2+ cells) + 3 × (% of 3+ cells)] [27]. The H score ≥ 10 was regarded as positive and ≥ 200 were considered high.

### *EGFR* gene copy number (GCN) by qPCR

Genomic DNA was extracted from frozen tumor samples or FFPE tumor sections using QIAamp Mini kit (Qiagen, Valencia, CA) according to the manufacturer's instructions. qPCR was carried out in a 20 μL reaction mixture containing genomic DNA, primers, and SYBR Premix Ex Taq II (TaKaRa, Cat#RR820A) (Supplementary Table 3). The primers for EGFR were 5′-GAATTCGGATGCAGAGCTTC-3′ for forward and 5′-GACATGCTGCGGTGTTTTC-3′ for reverse. The primers for internal control MTHFR (Methylene Tetrahydrofolate Reductase) were 5′-CCATCTTCCTGCTGCTGTAACTG-3′ for forward and 5′-GCCTTCTCTGCCAACTGTCC-3′ for reverse. The *EGFR* gene copy number was normalized to NCI-H441 cells. Samples with *EGFR* GCN ≥ 2.0 were further validated by EGFR FISH.

### *EGFR* gene amplification by FISH

The tumor specimens with *EGFR* GCN ≥ 2.0 identified by qPCR assay were further validated by FISH. In brief, 4 μm sections from FFPE samples were deparaffinized and rehydrated. Specimens were heated in the pre-treatment solution followed by proteolytic digestion using Pepsin (Dako FISH Accessary Kit, K5799). Tissue sections applied with *EGFR/CEP7* FISH probe (Vysis, Order NO. 01N35-020) were denatured at 73°C for 5 minutes and hybridized at 37°C overnight followed by a stringent wash with saline-sodium citrate containing Tween-20 (Dako FISH Accessory Kit, K5799). Finally, the specimens were mounted with fluorescence mounting medium (Invitrogen, P36935) containing 4′,6 diamidino-2-phenylindole (DAPI). The sections were visualized in a fluorescence microscope (Olympus BX53). The enumeration of *EGFR* gene and chromosome 7 was conducted in 50 tumor nuclei in each section by two independent observers. In case of discordance between the two observers, a third observer was involved. *EGFR* gene with ≥ 15 copies in ≥ 10% of cells or a gene/chromosome ratio per cell of ≥ 2.0 in homogenously stained regions was determined as *EGFR* gene amplification [28].

### Hot spot mutation detection of *EGFR*, *PIK3CA*, *K-Ras* and *B-Raf* genes

Hotspots mutations in exon 19, 20 and 21 of *EGFR* gene, exon 9 and 20 of *PIK3CA* gene, exon 2 and 3 of *K-Ras* gene and exon 11 and 15 of *B-Raf* gene were detected in tumor samples by the Genewiz Inc., using ABI3730XL sequence analyzer. The primers for detecting hot spot mutations in *EGFR*, *PIK3CA*, *K-Ras* and *B-Raf* are listed in Supplementary Table 4.

### EGFR kinase inhibition assay

EGFR kinase inhibition was determined using the Z´-LYTE^™^ kinase assay kit-Tyr 4 peptide (Invitrogen, PV3193). Briefly, EGFR kinase (Invitrogen, PV3872) was dissolved in a reaction buffer composed of 50 mM HEPES pH 7.5, 0.01% BRIJ-35, 10 mM MgCl_2_ and 1 mM EGTA. The final 10 μL of the kinase reaction mixture consisted of 5 ng kinase, 2 μM substrate peptide Try4, ATP and the test compounds theliatinib, gefitinib and erlotinib. ATP (10, 50, 200, 500, 800 and 1000 μM) was finally added to the reaction mixture to initiate the enzymatic reaction. The final concentration of DMSO in the assay was 2%. The reaction mixture was incubated at 25°C for 60 minutes in a 384 well plate. Then, 5 μL of Development Reagent B was added per well and incubated for further 60 minutes at 25°C. The fluorescent signal was read at emission wavelengths, 445 nm and 520 nm after excitation at 400 nm in a Victor3 multi label reader (PerkinElmer). The kinetic parameters (Ki, V_max_ and Km) were calculated with the Graphpad Prism software according to the Michaelis-Menten equation: *V* = Vmax × [S]/ (Km×(1+[I]/Ki)+ [S]). IC_50_ of thliatinib, gefitinib and erlotinib was calculated at different ATP concentrations using XLfit software (IDBS, Guildford, UK) [29].

### EGFR phosphorylation inhibition in tumor cells by DELFIA assay

EGF stimulated EGFR phosphorylation was determined in A431 human epidermoid carcinoma cells using the modified DELFIA assay. Briefly, A431 cells (1.3 × 10^4^ cells/well) were seeded overnight in a 96 well plate in 100 μL DMEM with 10% FBS. Then, the culture medium was removed and the cells were starved in FBS-free DMEM medium at 5% CO_2_ and 37°C for 24 h. Further, the cells were treated with 10 μL of the test compounds (theliatinib, gefitinib and erlotinib) at different concentrations (300~0.137 nM, 3 fold gradient dilution) for 60 minutes at 37°C and 5% CO_2_. The control cells were treated with 10 μL of FBS-free DMEM medium containing 5% DMSO (final concentration of DMSO was 0.5%). Cells were stimulated by 20 ng/mL recombinant human EGF (BIOSOURCE, PHG0064) for 45 minutes. Then, the cell supernatant was discarded and the cells were lysed with 100 μL DELFIA lysis buffer. The plates with cell lysates were kept at -80°C overnight. Then, the lysates were thawed on ice with gently mixing, and 20 μL of lysates were added into the assay plates (PerkinElmer, AAAND-0001), pre-coated with monoclonal anti-EGFR capture antibody (R&D, AF231), followed by incubation for 1h at room temperature. The phosphorylated EGFR (p-EGFR) in the lysates was detected with the detection mixture containing DELFIA Eu-N1 labeled anti-phospho-tyrosine antibody PT66 (PerkinElmer, Eu-PT66) and DELFIA enhancement solution for 1h at room temperature. Fluorescence signals were detected at 620 nm emission and 340 nm excitation by Victor3 multi label reader (PerkinElmer). All tested concentrations of theliatinib, gefitinib and erlotinib were repeated in duplicated wells.

### Cell survival assay

A431 cells (1 × 10^4^ cells/well) in exponential phase were seeded in duplicates in DMEM containing 10% FBS and incubated at 37°C and 5% CO^2^ overnight. Then, 10 μL of test compounds (theliatinib, gefitinib and erlotinib) at tested concentrations (10~0.005 μM, 3 fold gradient dilution) were added into each well with the final concentration of DMSO at 0.5%. The cells were incubated for 48 h followed by further incubation for 1h after adding 10 μL/well CCK-8 solution (Dojindo, CK-04-13). Cell survival was determined by measuring the optical density at 450 nm using Labsystems Multiskan K3 (Thermo Fisher Scientific Inc.).

### Western blot in PDECX tumor tissues for EGFR signaling inhibition

BALB/cASlac-nu/nu nude mice bearing PDECX1T0326 tumors were orally administered with theliatinib (15 mg/kg) or gefitinib (20 mg/kg). After 8 h, the animals were sacrificed and tumors were harvested. The tumors were homogenized in cold lysis buffer (Cell Signaling Technology, 9803) containing 1 mM PMSF (BIO BASIC INC., PB0425) and after centrifuging the lysates, the protein supernatant (containing 100 μg protein) were mixed with 5× SDS loading buffer and boiled at 100°C for 10 minutes and SDS-PAGE was performed (5% stacking gel at 80V for 20 minutes, then changed to 120V on 10% separating gel for 1h). Proteins were then transferred to nitrocellulose membranes (0.35A for 90 minutes), and incubated with the following antibodies individually: Phospho-EGFR (Tyr1068) (Invitrogen, 44788G), EGFR (Cell Signaling Technology, 2232), phospho-AKT (Cell Signaling Technology, 4060), AKT (Cell Signaling Technology, 9272), phospho-ERK (Thr202/Thr204) (Cell Signaling Technology, 4370), ERK (Cell Signaling Technology, 4695). After washing with the 1X TBST, the blots were incubated with the secondary IRDye 800-conjugated secondary antibody (LI-COR, 926-32211) and detected with chemiluminescence system.

## SUPPLEMENTARY MATERIALS TABLES







## Abbreviations

EC: esophageal cancer
ESCC: esophageal squamous cell carcinoma
EAC: esophageal adenocarcinoma
PDECX: patient derived esophagus cancer xenograft
IHC: immunohistochemistry
H&E: Hematoxylin and Eosin
FISH: fluorescence *in situ* hybridization
PCR: polymerase chain reaction
TKIs: tyrosine kinase inhibitors
FFPE: formalin fixed paraffin-embedded
HMPLACUC: Hutchison MediPharma Limited Animal Care and Use Committee
TV: tumor volume
TGI: tumor growth inhibition.
