# Supplementary material for: Anti-tumor efficacy of theliatinib in esophageal cancer patient-derived xenografts models with epidermal growth factor receptor (EGFR) overexpression and gene amplification
Source: Oncotarget. 2017 Apr 19;8(31):50832–44. doi: 10.18632/oncotarget.17243 (PMC5584209; doi:10.18632/oncotarget.17243)
Supplement: Supplementary file 1 [file oncotarget-08-50832-s001.pdf]

Anti-tumor efficacy of theliatinib in esophageal cancer patient-derived xenografts models with epidermal growth factor receptor (EGFR) overexpression and gene amplification

Supplementary Materials

Supplementary Table 1: EGFR IHC and GCN scores, and hot-spot mutation analysis for EGFR, PIK3CA, K-Ras and B-Raf genes in tumor samples from 70 Chinese esophageal cancer patients. See Supplementary_Table_1

Supplementary Table 2: Inhibition of kinases by theliatinib demonstrating its selectivity. See Supplementary_Table_2

Supplementary Table 3: The components of PCR reaction mixture

Reagent	Volume
SYBR Premix Ex Taq II (2×)	10 μL
PCR Forward Primer (10 μM)	0.5 μL
PCR Reverse Primer (10 μM)	0.5 μL
ROX Reference Dye II (50×)	0.4 μL
Template DNA	1 μL (< 250 ng)
ddH2O	7.6 μL
Total	20 μL

Supplementary Table 4: Primers for hot spot mutation detection in *EGFR, PIK3CA, K-Ras* and *B-Raf* genes

Gene range	Primer- Forward	Primer- Reverse
EGFR EXON 19	5′-CCCCAGCAATATCAGCCTTA-3′	5′-GCCAGTAATTGCCTGTTTCC-3′
EGFR EXON 20	5′-CATTCATGCGTCTTCACCTG-3′	5′-GATGGGACAGGCACTGATTT-3′
EGFR EXON 21	5′-GTCAGCAGCGGGTTACATCT-3′	5′-ACCCAGAATGTCTGGAGAGC-3′
PIK3CA EXON 9	5′-TGAAAATGTATTTGCTTTTTC-3′	5′-CAATGTAGTATGATTTTCC-3′
PIK3CA EXON 20	5′-TGGGGTAAAGGGAATCAAAA-3′	5′-ATCAAACCCTGTTTGCGTTT-3′
K-Ras EXON 2	5′-CTTAAGCGTCGATGGAGGAG-3′	5′-CCCTGACATACTCCCAAGGA-3′
K-Ras EXON 3	5′-GGTGCTTAGTGGCCATTTGT-3′	5′-CCTAGGTTTCAATCCCAGCA-3′
B-Raf EXON 11	5′-TTTTCTGTTTGGCTTGACTTGA-3′	5′-GAGTCCCGACTGCTGTGAAC-3′
B-Raf EXON 15	5′- AGCCCCAAAAATCTTAAAAGC-3′	5′-TGATTTTTGTGAATACTGGGAAC-3′
